# The impact of different radiology report formats on patient information processing: a systematic review

**DOI:** 10.1007/s00330-024-11165-w

**Published:** 2024-11-15

**Authors:** F. A. M. van der Mee, R. P. G. Ottenheijm, E. G. S. Gentry, J. M. Nobel, F. M. Zijta, J. W. L. Cals, J. Jansen

**Affiliations:** 1 Department of Family Medicine, Care and Public Health Research Institute, Maastricht University, Maastricht, The Netherlands; 2 Department of Radiology and Nuclear Medicine, Maastricht University Medical Center+, Maastricht, The Netherlands; 3 GROW Research Institute for Oncology and Reproduction, Maastricht University, Maastricht, The Netherlands

**Keywords:** Electronic health records, Health communication, Mental processes, Patient access to records, Radiology

## Abstract

**Background:**

Since radiology reports are primarily written for health professionals, patients may experience difficulties understanding jargon and terminology used, leading to anxiety and confusion.

**Objectives:**

This review evaluates the impact of different radiology report formats on outcomes related to patient information processing, including perception, decision (behavioral intention), action (actual health behavior), and memory (recall of information).

**Methods:**

PubMed, Web of Science, EMBASE, and PsycInfo were searched for relevant qualitative and quantitative articles describing or comparing ways of presenting diagnostic radiology reports to patients. Two reviewers independently screened for relevant articles and extracted data from those included. The quality of articles was assessed using the Mixed Methods Appraisal Tool.

**Results:**

Eighteen studies, two qualitative and sixteen quantitative, were included. Sixteen studies compared multiple presentation formats, most frequently traditional unmodified reports (*n* = 15), or reports with anatomic illustrations (*n* = 8), lay summaries (*n* = 6) or glossaries (*n* = 6). Glossaries, illustrations, lay summaries, lay reports or lay conclusions all significantly improved participants’ cognitive perception and perception of communication of radiology reports, compared to traditional reports. Furthermore, these formats increased affective perception (e.g., reduced anxiety and worry), although only significant for lay reports and conclusions.

**Conclusion:**

Modifying traditional radiology reports with glossaries, illustrations or lay language enhances patient information processing.

**Key Points:**

***Question*** *Identifying the impact of different radiology report formats on outcomes related to patient information processing to enhance patient engagement through online access to radiology reports.*

***Findings*** *Lay language summaries, glossaries with patient-oriented definitions, and anatomic illustrations increase patients’ satisfaction with and understanding of their radiology reports.*

***Clinical relevance*** *To increase patients’ satisfaction, perceived usefulness and understanding with radiology reports, the use of lay language summaries, glossaries with patient-oriented definitions, and anatomic illustrations is recommended. These modifications decrease patients’ unnecessary insecurity, confusion, anxiety and physician consultations after viewing reports.*

## Introduction

Nowadays, most patients have real-time access to their own online patient portal, including diagnostic radiology results [1–4]. Potential benefits of patients’ online access to their test results include improving patients’ knowledge, patient engagement, and self-efficacy. It may also improve communication with their physician and streamline consultations [1, 5–8]. At the same time, radiology reports tend to be written for physicians and are difficult to understand for patients, leading to misinterpretation of information, insecurity, confusion, anxiety, and stress [9–11]. This is especially true when patients read their radiology reports prior to their physician consultation. Studies have shown that a poor understanding of radiology test results increases the number of consultations, and may result in unnecessary follow-up testing, emergency department visits, and even hospital admissions [12, 13]. As healthcare evolves toward a more patient-centered approach, the utilization of online patient portals will continue to increase. Therefore, it is of great importance to be aware of the barriers patients experience when accessing and interpreting radiology reports [2, 4, 14–17].

Radiologists and referring physicians are aware that direct patient access to radiology reports can lead to misunderstandings, due to the specialized jargon and technical terminology these reports often contain [18–21]. This may impede patient comprehension, especially if reports are longer [13, 22–27]. Several initiatives, such as introducing lay language or illustrations to the radiological report, have been proposed to improve patient understanding [15, 28, 29]. However, the effect of these variations in radiology report formats on patient information processing has not been compared across different studies.

To help systematically synthesize the evidence, a recent taxonomy distinguishes four types of outcomes related to patient information processing: perception, decision/behavioral intention, action/actual health behavior, and memory [30, 31]. Perception is related to the cognitive and affective meaning that patients derive from information (e.g., feelings about the information that is communicated, such as worry, classification of information as ‘normal’ or ‘abnormal’, preferences for certain ways of communicating information). Decision/behavioral intention is the step before health behavior (e.g., intention to go online to search for more information, intention to consult a doctor). Action is the actual health behavior (e.g., following the doctor’s recommendations), and memory is what patients recall of the information they received.

We aimed to conduct a systematic review of the literature to evaluate the effect of different radiology report formats on patient information processing, specifically perception, decision, action, and memory.

## Methods

This review was reported in accordance with the Preferred Reporting Items for Systematic Reviews and Meta-Analyses (PRISMA) (Appendix 1) [32]. A protocol for this review was not previously registered.

### Search strategy

Four databases (PubMed, Web of Science, EMBASE, and PsycInfo) were searched up to September 30th, 2023. The first author (F.M.—medical doctor; general practitioner in training and PhD candidate) developed a search for each database, containing both thesaurus and free text terms. In EMBASE, a filter was applied to remove preprint records and exclude MEDLINE citations, since the latter were already covered by the PubMed search. The complete reproducible search can be found in Appendix 2. Forward and backward snowballing was performed by two authors (F.M. and E.G.—senior medical student) [33] and experts in the field of radiology communication (J.N. and F.Z.—radiologists; J.J.—behavioral scientist in the field of health communication and medical decision making) were asked to find relevant missed articles.

### Study selection and eligibility criteria

After removing duplicates, titles and abstracts were independently screened on relevance by two authors (F.M. and E.G.) using Endnote 20 [34] and Covidence [35]. All original research papers describing or comparing ways of presenting diagnostic radiology test results to patients were included if available in English or Dutch. In addition, full texts were screened, and papers were only included when the format of the radiology report was clearly described, when describing original research not related to screening, and when at least one outcome related to patient information processing was measured [31]. Disagreements were resolved by discussion or screening by a third reviewer (R.O.—medical doctor and PhD; general practitioner with special interest in musculoskeletal disorders). Cohen’s kappa was calculated to determine the level of agreement between the two reviewers [36].

### Data extraction

Two authors (F.M. and E.G.) independently extracted relevant data of eligible studies using a form that was developed by the multidisciplinary team and used in a previous study [37]. Year of publication, country, study design, participant characteristics, and study inclusion and exclusion criteria were assessed. Furthermore, radiology report format, type of radiology test(s), and whether real or hypothetical data was used were extracted.

### Outcome measures

**Fig. 1:**
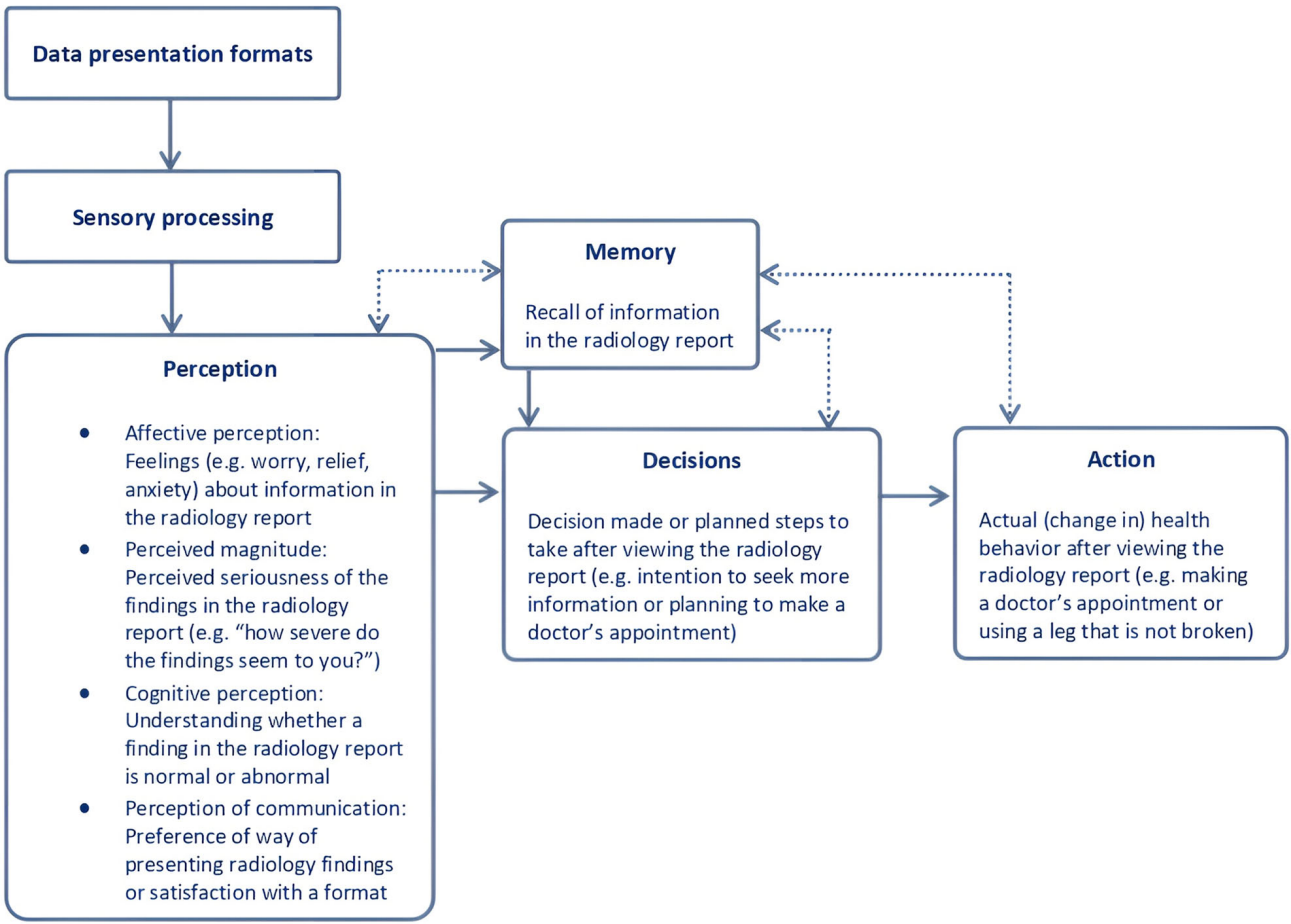
Visualization of patient outcome measure categories based on Ancker’s taxonomy of human information processing

Outcome measures were extracted and classified independently by two authors (F.M. and E.G.) into the following categories of information processing: perception, decision/behavioral intention, action/actual health behavior, and memory. Perception was further subdivided into four subcategories: affective perception, perceived magnitude, cognitive perception, and perception of communication (Fig. 1) [31, 38, 39].

### Quality assessment

To evaluate quality and risk of bias, the Mixed Methods Appraisal Tool (MMAT) was used [40]. The MMAT is specifically designed to assess studies with diverse methodologies and study designs, including qualitative and quantitative studies [41]. The tool contains five criteria per study design. Two reviewers (F.M. and E.G.) discussed both data and quality extraction until consensus was reached. The quality assessment tool (MMAT) evaluates studies based on five criteria per study design, with each criterion allocated a score of 20% if present [40].

### Data synthesis

To integrate both qualitative and quantitative findings, a convergent integrated approach was used, combining extracted data from qualitative and quantitative studies [42, 43]. As recommended by the Joanna Briggs Institute Mixed Methods Review Methodology Group, quantitative data was qualitized, as this is less error-prone than attributing numerical values to qualitative data, resulting in a narrative interpretation of quantitative results [42]. In this review, when describing findings as “significant”, we refer to statistically significant effects as reported in the cited articles.

## Results

**Fig. 2:**
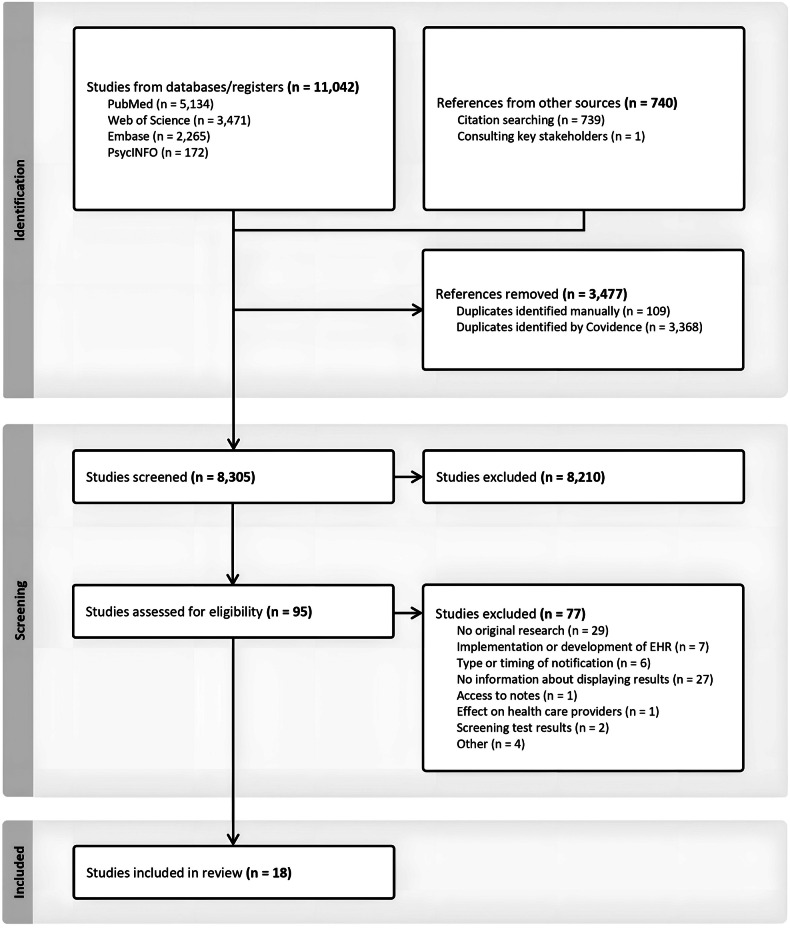
Flow chart of the study selection process

The initial search identified 11,782 references. After removing duplicates (*n* = 3477) and screening for relevance, 18 articles were included in this review (Fig. 2). Cohen’s kappa for interrater reliability was 0.61 for title and abstract screening and 0.81 for full-text screening, indicating, respectively, a moderate and strong agreement [36]. The third reviewer was consulted for 6.3% of the articles in the full-text screening (*n* = 6/95).

### Study characteristics

Two qualitative and 16 quantitative studies were included (total *n* = 18), published between 2012 and 2023, the majority conducted in the United States of America (*n* = 16). The total sample size of all included studies was 23,997 participants, ranging from 13 to 16,735 participants. Sex distribution was almost equal among the articles reporting this characteristic (54.5% female) (*n* = 12). Eleven studies reported educational level, with 77.5% of the participants reporting higher education (defined as college degree or higher). The overall characteristics of the included studies and populations are summarized in Table 1.

**Table 1:** Study and population characteristics of all included studies (*n* = 18)

Author (year)	Country	Study design	Sample *N*	Population characteristics				Aim of study
				Sample	Sex (% female)	Age (in years)	Education (% low/middle/high education)^a^	
Alarifi et al [51]	USA	Randomized controlled trial	616	Amazon MTurk	41.4	40.9% < 20–29; 44.6% 30–49; 14.5% > 50	0/37.5/62.5	To compare original MRI lumbar spine reports with patient-friendly reports and examine patient perceptions of understanding, cosmetic appearance, perceived ease of use, acceptance, and preference.
Bossen et al [44]	USA	Cross-sectional study	100	Real patients	59.0	Mean 51 (SD 16, range 21–80)	Mean 15 years of education (SD 2.9) (range 4–24)	To determine whether rewording MRI reports in understandable, more dispassionate language will result in better patient ratings of emotional response, satisfaction, usefulness, and understanding.
Cho et al [52]	USA	Cross-sectional study	203	Amazon MTurk		17.7% 18–25; 47.8% 26–35; 14.8% 36–45; 12.3% 46–55; 3.9% 56–65; 3.5% > 66	0/34.5/65.5	To assess patient comprehension of five radiology reporting templates.
Cook et al [15]	USA	Cross-sectional study	22	Real patients				To evaluate a web-based interface presenting knee MRI reports with annotations that include patient-oriented definitions, anatomic illustrations, and hyperlinks to additional information.
Dabrowiecki et al [54]	USA	Survey	5155	Amazon MTurk	54.5	17.1% 18–24; 41.9% 25–34; 21.1% 35–44; 10.9% 45–54; 6.5% 55–65; 2.5% > 65	0/17.5/82.5	To assess public preferences and perceptions regarding negative chest radiograph reports.
Dy et al [67]	USA	Randomized controlled trial	44	Real patients				To test a patient-centered radiology report for pediatric renal ultrasounds to enhance parental understanding of the report.
Gunn et al [27]	USA	Cross-sectional study	104	Real patients	55.8	30% 51–60 Other age groups not stated	0/37.0/63.0	To describe experiences receiving structured feedback from patients on actual radiology reports as a means of improving reporting practices.
Johnson et al [56]	USA	Survey	53	Real patients	45.3	Mean 57 (range 22–82)	3.8/30.2/66.0	To determine the patient-preferred timing characteristics of a system for online patient access to radiologic reports, and patient resource needs and preferences after exposure to these reports.
Kadom et al [29]	USA	Survey	199	Amazon MTurk	42.4	51.5% 20–30; 41.4% 31–50; 7.1% 51–75	0/17.2/82.8	To assess whether Information Reporting and Data Systems would decrease patients’ anxiety, increase calls to providers, and elucidate whether patients desire these messages to be included in radiology reports.
Kemp et al [53]	USA	Cross-sectional study	16,735	Real patients				To evaluate patient use and experience with patient-centered radiology reports provided via a radiology-specific patient portal in an outpatient setting.
Norris et al [45]	USA	Survey	299	Real patients	69.2	1.3% 18–24; 10.4% 25–34; 13.4% 35–44; 16.4% 45–54; 27.1% 55–64; 31.4% > 65	1/14.8/51.5 Other education level not stated	To understand patient experiences, opinions, and actions taken after viewing their radiology images via an electronic patient portal.
Perlis et al [48]	Canada	Qualitative study	15	Real patients	0	Mean 67		To create a patient-centered prostate MRI report to give patients a better understanding of their clinical condition.
Perlis et al [28]	Canada	Randomized controlled trial	40	Real patients	0	Mean 65.3 (SD 7.3, range 46–77)	0/15.8/84.2	To design a patient-centered prostate MRI report and to test whether these reports improve patient knowledge and experience.
Recht et al [46]	USA	Cross-sectional study	101	Real patients				To create patient-centered video radiology reports using simple-to-understand language and annotated images and to assess the effect of these reports on patients’ experience and understanding of their imaging results.
Short et al [47]	USA	Randomized controlled trial	193	Amazon MTurk	100	7.3% 18–22; 55.4% 25–44; 34.2% 45–64; 3.1% > 65		To evaluate the effectiveness of a patient-centered web-based interactive mammography report.
Wieland et al [50]	USA	Randomized controlled trial	85	Real patients	57.6	3.5% < 30; 2.4% 30–44; 30.6% 45–64; 50.6% 65–79; 12.9% > 80	12.9/64.7/14.1 Other education levels not stated	To assess oncology patients’ confidence and accuracy in interpreting radiology reports either with or without layman translations.
Woo et al [49]	USA	Survey	20	Real patients		Median 50		To evaluate whether implementing structured reporting based on Ovarian-Adnexal Reporting and Data System MRI in women with sonographically indeterminate adnexal masses improves communication between radiologists, referrers, and patient/caregivers.
Zhang et al [55]	USA	Qualitative study	13	Volunteers	53.8	7.7% 18–25; 46.2% 26–49; 46.2% 50–64	0/23.1/77.0	To understand patients’ perceptions and acceptance of using artificial intelligence technology to interpret their radiology reports.

*Amazon MTurk* Amazon Mechanical Turk, *MRI* magnetic resonance imaging, *SD* standard deviation, *USA* United States of America

^a^ Low education: primary school. Middle education: secondary/high/trade school, some college. High education: 4-year/college/associate/university/undergraduate/Bachelor’s/Master’s/advanced/professional/doctorate degree

Radiology tests included magnetic resonance imaging (*n* = 11), computed tomography (*n* = 6), x-ray (*n* = 5), and ultrasound (*n* = 5) examinations. In total, participants in 12 of the 18 studies were real patients, the remainder used healthy volunteers mostly through the Amazon Mechanical Turk (Amazon MTurk) platform (*n* = 5). Most of the studies used mock (or hypothetical) test results (*n* = 12). Sixteen studies compared multiple presentation formats, most frequently traditional narrative reports without modifications (*n* = 15), reports with anatomic illustrations (*n* = 8), lay summaries (*n* = 6) or glossaries (*n* = 6) (Table 2).

**Table 2:**
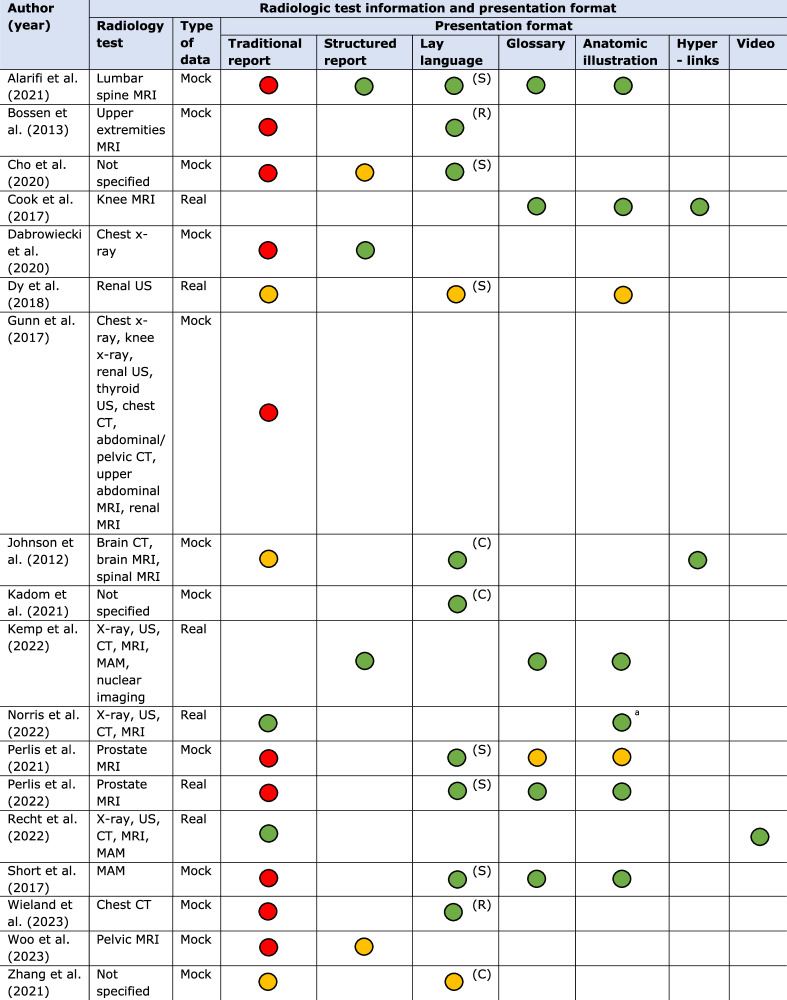
Radiologic test characteristics and presentation format used in all included studies (*n* = 18)

Explanation of colors: green, format with evident preference; yellow, format with slight preference; red, format with the least preference

Explanation of the presentation formats used: traditional report, original narrative radiology report without any modifications. Structured report, report with organized structure by describing findings separately using subheadings instead of describing all findings in one paragraph. Lay language report (R), whole report translated into lay language. Lay summary (S), only a summary of the report in lay language. Lay conclusion (C), only a conclusion of the findings in lay language. Glossary, patient-oriented definitions for any medical terminology in the report. Anatomic illustration, illustrations to explain the anatomy described in the report. Hyperlinks, hyperlinks to related, external websites with additional information about the examination or results. Video, report including a video with spoken explanations of the findings

*MRI* magnetic resonance imaging, *US* ultrasound, *CT* computed tomography, *MAM* mammography

^a^ Original radiology images were provided to patients instead of illustrations

### Quality assessment

Both qualitative articles scored 100%, indicating excellent quality. Scores for quantitative articles (*n* = 16) varied between 20% and 80% (Appendix 3). Factors contributing to lower scores included sample unrepresentativeness by not including real patients, utilization of non-validated questionnaires, unclear blinding procedures, and not accounting for confounders in the analysis.

### Outcome measures

All 18 studies assessed at least one subcategory of patient information processing outcomes, with 12 studies examining multiple (sub)categories. Perception was the most frequently studied, in particular perception of communication and cognitive perception (both *n* = 14), followed by affective perception (*n* = 6) and perceived magnitude (*n* = 3) (Table 3, Fig. 1). Additionally, decision and action were examined by three and two studies, respectively. None of the studies studied memory as outcome measure.

**Table 3:** The outcomes assessed in all included studies (*n* = 18)

Author (reference)	Perception				Decision	Action	Memory
	Affective perception	Perceived magnitude	Cognitive perception	Perception of communication	Behavioral intention	Health behavior	Verbatim recall
Alarifi et al [51]			x	x			
Bossen et al [44]	x		x	x			
Cho et al [52]			x				
Cook et al [15]			x	x			
Dabrowiecki et al [54]			x		x		
Dy et al [67]			x	x			
Gunn et al [27]			x				
Johnson et al [56]				x	x		
Kadom et al [29]	x	x		x	x		
Kemp et al [53]			x	x			
Norris et al [45]	x		x	x		x	
Perlis et al [48]		x		x			
Perlis et al [28]	x		x	x		x	
Recht et al [46]	x		x	x			
Short et al [47]	x		x	x			
Wieland et al [50]			x				
Woo et al [49]		x	x	x			
Zhang et al [55]				x			

### Perception—affective perception

Six studies examined participants’ emotional responses while viewing and interpreting their radiology results [28, 29, 44–47]. Participants were significantly less anxious, calmer, and felt more in control after reading hypothetical lay language reports, compared to traditional reports [44]. Adding only a lay language conclusion or communicating results via a video report also resulted in decreased anxiety levels for a majority of the studied participants [29, 46]. Providing participants their actual radiology images resulted in more reassurance and less confusion among the majority of participants after viewing these images [45]. However, no significant differences in anxiety levels were found between participants who received reports with lay summaries, glossaries and anatomic illustrations compared to those who received traditional reports, although reduced anxiety was a trend in all these studies (Table 4) [28, 47].

**Table 4:**
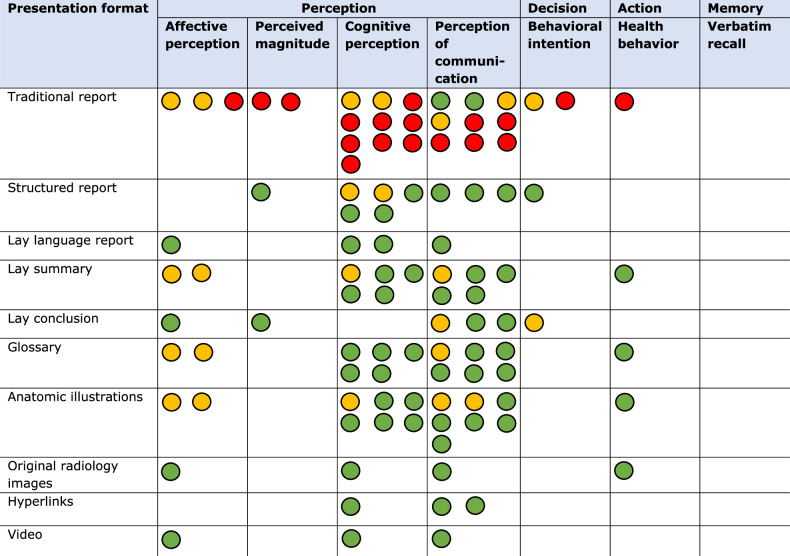
Presentation formats used in all included studies and their effects on the different outcome measures used

Each circle represents one study in which a presentation format was used to examine the corresponding outcome

Explanation of colors: green, significant positive effect of presentation format on outcome; yellow, slightly positive, but not significant, effect of presentation format on outcome; red, significant negative effect of presentation format on outcome

### Perception—perceived magnitude

To determine the perceived magnitude of risk associated with the information in the radiology appeared to be difficult for participants. In one study among patients with prostate cancer, participants stated that summarizing the results in lay language increased their perception of their overall cancer risk [48]. The use of lay language conclusions increased participants’ sense of urgency after viewing results that required follow-up with referring physicians [29]. Radiology reports with a structured format helped participants to better understand the likelihood of the presence of cancer according to the findings in the report [49].

### Perception—cognitive perception

Fourteen studies examined participants’ cognitive perception of radiology reports. In one study of hypothetical traditional radiology reports, participants reported limited understanding of these reports [27]. Problems included the length of the report and difficulties in understanding medical terminology [27, 50]. Overall, participants’ self-reported understanding and ease of reading improved when lay language reports [44, 50], lay language summaries [28, 51, 52], or glossaries with patient-oriented definitions and anatomic illustrations were provided [15, 28, 47, 51, 53]. Using hyperlinks [15], access to original radiology images [45], or video reports [46] also increased participants’ subjective understanding. The combination of hypothetical structured reports with lay summaries resulted in a significant increase in participants’ objective understanding, compared with traditional reports [51, 52]. Studies examining the effect of hypothetical structured reports compared to traditional reports demonstrated inconsistent findings. One study showed a significantly better understanding of structured reports [54], while another study found no significant differences (Table 4) [49].

### Perception—perception of communication

Participants identified lay and concise language as the most important component of accessible radiology reports, followed by the addition of glossaries and anatomic illustrations [48]. Overall, a significant increase in participants’ satisfaction and perceived usefulness of radiology reports was found when providing both real and hypothetical reports with a lay language summary of the findings [28, 47, 51], or with glossaries and anatomic illustrations [15, 28, 51, 53]. This effect was also found when reports were completely written in lay language [44] or when structured reports were provided [49, 51, 53]. Lay conclusions appeared to be helpful for understanding hypothetical radiology reports for the majority of participants [29, 55, 56]. The addition of hyperlinks [15, 56], access to original radiology images [45], or the use of video reports [46] were all considered helpful for participants compared to traditional reports.

### Decision/behavioral intention

Three studies assessed behavioral intention, especially participants’ intention to contact their physician after viewing hypothetical radiology test results [29, 54, 56]. Adding a lay language conclusion reduced the intention to contact healthcare professionals in case of no concerning test results [29]. Furthermore, participants with normal test results were significantly less likely to contact their physician when presented with structured reports, compared with traditional reports (Table 4) [54].

### Action/actual health behavior

One study looked at a patient-centered radiology report of real data, consisting of a lay language summary, glossary with patient-oriented definitions, and anatomic illustrations [28]. Participants considered patient-centered reports significantly more helpful in discussing follow-up steps with physicians and communicating results to family and friends than standard reports (Table 4) [28]. Another study examined the effects of providing original radiology images to real patients. The majority of patients indicated that viewing their images online allowed them to better follow their physician’s recommendations [45].

### Memory

None of the included studies assessed memory as outcome measure.

## Discussion

This review demonstrates that modification of traditional radiology reports is important to enhance patient information processing. Due to limited data, little is known about the impact of presentation formats of radiology reports on the last three steps of the human information processing cascade; decision/behavioral intention, action/actual health behavior, and memory (Fig. 1). The first step of information processing, perception, has been studied extensively in the included studies. Overall, the addition of glossaries with patient-oriented definitions, anatomic illustrations, lay language summaries, lay language reports or lay language conclusions resulted in a significant increase in patients’ satisfaction, perceived usefulness (perception of communication) and subjective and objective understanding (cognitive perception) of radiology reports. In addition, the use of lay language reports or conclusions also significantly reduced patients’ anxiety (affective perception), and lay conclusions increased patients’ sense of urgency (perceived magnitude), compared to traditional reports. Looking only at the studies that involved real reports (*n* = 6) and patients (*n* = 12), the use of lay language summaries, glossaries, and illustrations were the most important formats to enhance participants’ perception of communication (satisfaction, usefulness), and cognitive perception (both objective and subjective understanding).

When hypothetical scenarios are used, participants lack personal relevance of test results and are not able to sufficiently value these reports. Therefore, findings of studies using hypothetical data may not reflect how participants would respond in real life to their own personal health information [57–59]. Several studies describe this phenomenon of “hypothetical bias”, in which participants overstate their preferences or behavior when the situation is hypothetical, compared to when they are exposed to real situations [60–63].

Five studies used Amazon MTurk as crowdsourcing platform. In general, MTurk users are younger and have above-average computer literacy, compared to the general population [64–66]. In addition, among the included articles reporting educational level, 77.5% of the participants reported a higher education level, which is higher than in the general population. Both factors may limit the generalizability of the findings of this review to the general population.

Another limitation of this review is the heterogeneity in outcome measures in the included studies. Included articles varied widely in study design, radiology report formats and outcome measures used. Two authors (F.M. and E.G.) independently extracted and classified all outcome measures of all studies in the four categories of information processing. As a result, each category contains different types and assessment methods of outcome measures, making comparison of the findings challenging. Appendix 4 provides an overview of the different outcome measures included in every patient information processing category. Furthermore, we included both quantitative and qualitative studies in our review. We followed the recommendations of the Joanna Briggs Institute Mixed Methods Review Methodology Group to transform the data. This resulted in qualitalizing quantitative data, as this is less error-prone than contributing numerical values to qualitative data. However, comparison of outcome measures is difficult in a mixed methods systematic review.

Eleven studies examined the effect of lay language reports, summaries or conclusions. However, seven of these studies lacked a definition of what is considered lay language. The other four studies considered different reading levels as lay language, varying from below fifth-grade to below eighth-grade level, in which ‘grade’ refers to years of education [29, 44, 50, 67]. These reading levels correspond with current guidelines recommending patient information should not exceed sixth- to eighth-grade reading level [19, 68–70]. An interesting finding concerning a possible increase in compliance was seen when patients could view their own images [45]. This is important because an image can “say more than a thousand words”. When these images are accompanied by an understandable report it can help the process of knowledge transfer. Especially if the report is aimed at a specific receiver, being the general practitioner, the referring physician, or the patient. Hence, for physicians the combined information is available, why should it not be available for the patient—perhaps in a simple format by means of an illustration?

Radiology tests requested by hospital physicians are more likely to reveal clinically significant findings and utilize more sophisticated and detailed test protocols compared to those requested by primary care physicians. Therefore, patients with known diseases within the hospital context may perceive the information differently compared to patients undergoing a radiology test referred by primary care physicians, with no previously known disease. As a result, these patients might prefer different presentation formats of radiology reports based on the referring specialty. This potential difference could be a focus of future research.

In addition, the use of artificial intelligence (AI) in medical healthcare is increasing, and therefore it is important to investigate how AI can enhance the sensory processing concerning the knowledge transfer of results of the radiological examination as well. Especially with the introduction of image generation tools and large language models (LLMs) it might be possible to fine-tune this process by adjusting the way of presenting the output to the knowledge level of the receiver. LLMs, in particular, can be useful in the field of information transfer, such as explaining the content of the radiological report or impressions in lay language, or in providing additional information. However, it is important to recognize that LLMs do “hallucinate”, meaning that information can be incorrectly presented to the receiver if the input information is incorrect or un-checked [71, 72]. Therefore, when using LLMs, it should be done in a setting that is convenient for both the physician and the patient. Thereby ensuring that they both understand that the output of AI may not always be accurate. To warrant a reliable output, it is crucial that processing takes place in a well-regulated setting. Of course, this may seem futuristic and regulations (e.g., data sharing policies) nowadays are not covering this possibility yet. Still, we should try to also focus on possibilities of how AI can help the patient understand their physical situation, especially in times when LLMs tend to evolve significantly.

This review demonstrates that modifying the radiology reporting process, traditionally created for physician-to-physician communication, may result in a shift toward a more patient-centered approach, prioritizing readability and comprehensibility and thereby ultimately enhancing patient information processing. Yet, the effects of changes in the reporting process, workload and technical requirements for the radiologist and their equipment were outside the scope. However, it is clear that altering the reporting process would have a significant impact on the radiological workflow. Still, we should focus on the potential of structured reporting, standardization and AI like LLMs to enhance information processing by means of modifying the radiology report [73–76].

To our knowledge, this is the first review to systematically synthesize available data on the effect of different radiology report formats on outcomes related to patient information processing. A multidisciplinary team of general practitioners, behavioral scientists, and radiologists was involved in this review, which is one of its strengths. Eligibility screening was performed by two independent authors, as well as data extraction using a published taxonomy to enable comparison of different studies.

As the results are narrative, a limitation of this review is the potential risk of bias introduced by the authors when describing the findings.

Overall, this systematic review highlights the importance of modifying traditional radiology reports to enhance patient information processing. To increase patients’ satisfaction, perceived usefulness and understanding of radiology reports, the use of lay language summaries, glossaries with patient-oriented definitions, and anatomic illustrations is recommended. Our results furthermore suggest that these formats not only help patients better understand their radiology reports and increase satisfaction, they may also reduce anxiety, and prevent extra physician consultations.

## Supplementary information

ELECTRONIC SUPPLEMENTARY MATERIAL

## Abbreviations

AI: Artificial intelligence
Amazon MTurk: Amazon Mechanical Turk
LLMs: Large language models
MMAT: Mixed Methods Appraisal Tool
MRI: Magnetic resonance imaging
SD: Standard deviation
USA: United States of America

## References

[1] Bhavnani V, Fisher B, Winfield M, Seed P (2011) How patients use access to their electronic GP record—a quantitative study. Fam Pract 28:188–194 21084568 10.1093/fampra/cmq092

[2] Lee CI, Langlotz CP, Elmore JG (2016) Implications of direct patient online access to radiology reports through patient web portals. J Am Coll Radiol 13:1608–1614 27888949 10.1016/j.jacr.2016.09.007

[3] Ralston JD, Carrell D, Reid R, Anderson M, Moran M, Hereford J (2007) Patient web services integrated with a shared medical record: patient use and satisfaction. J Am Med Inform Assoc 14:798–806 17712090 10.1197/jamia.M2302 PMC2213480

[4] Miles RC, Hippe DS, Elmore JG, Wang CL, Payne TH, Lee CI (2016) Patient access to online radiology reports: frequency and sociodemographic characteristics associated with use. Acad Radiol 23:1162–1169 27287715 10.1016/j.acra.2016.05.005

[5] Woods SS, Schwartz E, Tuepker A et al (2013) Patient experiences with full electronic access to health records and clinical notes through the My HealtheVet Personal Health Record Pilot: qualitative study. J Med Internet Res 15:e65 23535584 10.2196/jmir.2356 PMC3636169

[6] Bartlett C, Simpson K, Turner AN (2012) Patient access to complex chronic disease records on the Internet. BMC Med Inform Decis Mak 12:87 22867441 10.1186/1472-6947-12-87 PMC3438097

[7] Baun C, Vogsen M, Nielsen MK, Høilund-Carlsen PF, Hildebrandt MG (2020) Perspective of patients with metastatic breast cancer on electronic access to scan results: mixed-methods study. J Med Internet Res 22:e15723 32039819 10.2196/15723 PMC7055828

[8] Fisher B, Bhavnani V, Winfield M (2009) How patients use access to their full health records: a qualitative study of patients in general practice. J R Soc Med 102:539–544 19966130 10.1258/jrsm.2009.090328 PMC2789021

[9] Garrido T, Jamieson L, Zhou Y, Wiesenthal A, Liang L (2005) Effect of electronic health records in ambulatory care: retrospective, serial, cross sectional study. BMJ 330:581 15760999 10.1136/bmj.330.7491.581 PMC554034

[10] Pillemer F, Price RA, Paone S et al (2016) Direct release of test results to patients increases patient engagement and utilization of care. PLoS One 11:e0154743 27337092 10.1371/journal.pone.0154743 PMC4919031

[11] Sung S, Forman‐Hoffman V, Wilson MC, Cram P (2006) Direct reporting of laboratory test results to patients by mail to enhance patient safety. J Gen Intern Med 21:1075–1078 16836627 10.1111/j.1525-1497.2006.00553.x PMC1831617

[12] Palen TE, Ross C, Powers JD, Xu S (2012) Association of online patient access to clinicians and medical records with use of clinical services. J Am Med Assoc 308:2012–2019 10.1001/jama.2012.14126 23168824

[13] Rosenkrantz AB (2017) Differences in perceptions among radiologists, referring physicians, and patients regarding language for incidental findings reporting. AJR Am J Roentgenol 208:140–143 27657356 10.2214/AJR.16.16633

[14] Tieu L, Schillinger D, Sarkar U et al (2017) Online patient websites for electronic health record access among vulnerable populations: portals to nowhere? J Am Med Inform Assoc 24:e47–e54 27402138 10.1093/jamia/ocw098 PMC6080722

[15] Cook TS, Oh SC, Kahn Jr CE (2017) Patients’ use and evaluation of an online system to annotate radiology reports with lay language definitions. Acad Radiol 24:1169–1174 28433519 10.1016/j.acra.2017.03.005

[16] Bruno MA, Petscavage-Thomas JM, Mohr MJ, Bell SK, Brown SD (2014) The “open letter”: radiologists’ reports in the era of patient web portals. J Am Coll Radiol 11:863–867 24836272 10.1016/j.jacr.2014.03.014

[17] Arnold CW, McNamara M, El-Saden S, Chen S, Taira RK, Bui AA (2013) Imaging informatics for consumer health: towards a radiology patient portal. J Am Med Inform Assoc 20:1028–1036 23739614 10.1136/amiajnl-2012-001457 PMC3822110

[18] Johnson AJ, Frankel RM, Williams LS, Glover S, Easterling D (2010) Patient access to radiology reports: what do physicians think? J Am Coll Radiol 7:281–289 20362944 10.1016/j.jacr.2009.10.011

[19] Martin-Carreras T, Cook TS, Kahn JrCE (2019) Readability of radiology reports: implications for patient-centered care. Clin Imaging 54:116–120 30639521 10.1016/j.clinimag.2018.12.006

[20] Wallis A, McCoubrie P (2011) The radiology report—are we getting the message across? Clin Radiol 66:1015–1022 21788016 10.1016/j.crad.2011.05.013

[21] Farmer CI, Bourne AM, O’Connor D, Jarvik JG, Buchbinder R (2020) Enhancing clinician and patient understanding of radiology reports: a scoping review of international guidelines. Insights Imaging 11:1–10 32372369 10.1186/s13244-020-00864-9 PMC7200955

[22] Farmer C, O’Connor DA, Lee H et al (2021) Consumer understanding of terms used in imaging reports requested for low back pain: a cross-sectional survey. BMJ Open 11:e049938 34518265 10.1136/bmjopen-2021-049938 PMC8438839

[23] McCaffery K, Nickel B, Moynihan R et al (2015) How different terminology for ductal carcinoma in situ impacts women’s concern and treatment preferences: a randomised comparison within a national community survey. BMJ Open 5:e008094 26525720 10.1136/bmjopen-2015-008094 PMC4636630

[24] Mityul MI, Gilcrease-Garcia B, Searleman A, Demertzis JL, Gunn AJ (2018) Interpretive differences between patients and radiologists regarding the diagnostic confidence associated with commonly used phrases in the radiology report. AJR Am J Roentgenol 210:123–126 29023151 10.2214/AJR.17.18448

[25] Alarifi M, Patrick T, Jabour A, Wu M, Luo J (2021) Understanding patient needs and gaps in radiology reports through online discussion forum analysis. Insights Imaging 12:1–9 33871753 10.1186/s13244-020-00930-2 PMC8055745

[26] Bosmans JM, Weyler JJ, Parizel PM (2009) Structure and content of radiology reports, a quantitative and qualitative study in eight medical centers. Eur J Radiol 72:354–358 18667286 10.1016/j.ejrad.2008.06.023

[27] Gunn AJ, Gilcrease-Garcia B, Mangano MD, Sahani DV, Boland GW, Choy G (2017) JOURNAL CLUB: structured feedback from patients on actual radiology reports: a novel approach to improve reporting practices. AJR Am J Roentgenol 208:1262–1270 28402133 10.2214/AJR.16.17584

[28] Perlis N, Finelli A, Lovas M et al (2022) Exploring the value of using patient-oriented MRI reports in clinical practice—a pilot study. Support Care Cancer 30:6857–6876 35534628 10.1007/s00520-022-07108-0

[29] Kadom N, Tamasi S, Vey BL et al (2021) Info-RADS: adding a message for patients in radiology reports. J Am Coll Radiol 18:128–132 33068534 10.1016/j.jacr.2020.09.049

[30] Wickens CD, Helton WS, Hollands JG, Banbury S (2021) Engineering psychology and human performance. Routledge, New York

[31] Ancker JS, Benda NC, Sharma MM, Johnson SB, Weiner S, Zikmund‐Fisher BJ (2022) Taxonomies for synthesizing the evidence on communicating numbers in health: goals, format, and structure. Risk Anal 42:2656–2670 35007354 10.1111/risa.13875 PMC10241486

[32] Page MJ, McKenzie JE, Bossuyt PM et al (2021) The PRISMA 2020 statement: an updated guideline for reporting systematic reviews. Int J Surg 88:105906 33789826 10.1016/j.ijsu.2021.105906

[33] Wohlin C (2014) Guidelines for snowballing in systematic literature studies and a replication in software engineering. In: Proceedings of the 18th International Conference on Evaluation and Assessment in Software Engineering, London. Association for Computing Machinery, pp 1–10

[34] The EndNote Team (2013) EndNote. EndNote 20 ed. Clarivate, Philadelphia

[35] Covidence systematic review software. Veritas Health Innovation, Melbourne. Available via www.covidence.org. [Accessed 20 February 2024]

[36] McHugh ML (2012) Interrater reliability: the kappa statistic. Biochem Med 22:276–282 PMC3900052 23092060

[37] van der Mee FAM, Schaper F, Jansen J, Bons JAP, Meex SJR, Cals JWL (2024) Enhancing patient understanding of laboratory test results: systematic review of presentation formats and their impact on perception, decision, action, and memory. J Med Internet Res 26:e53993 39133906 10.2196/53993 PMC11347896

[38] Becker MH (1974) The health belief model and personal health behavior. Health Educ Monogr 2:324–508

[39] Witte K (1992) Putting the fear back into fear appeals: the extended parallel process model. Commun Monogr 59:329–349

[40] Hong QN, Fàbregues S, Bartlett G et al (2018) The mixed methods appraisal tool (MMAT) version 2018 for information professionals and researchers. EFI 34:285–291

[41] Pace R, Pluye P, Bartlett G et al (2012) Testing the reliability and efficiency of the pilot Mixed Methods Appraisal Tool (MMAT) for systematic mixed studies review. Int J Nurs Stud 49:47–53 21835406 10.1016/j.ijnurstu.2011.07.002

[42] Stern C, Lizarondo L, Carrier J et al (2021) Methodological guidance for the conduct of mixed methods systematic reviews. JBI Evid Implement 19:120–129 34061049 10.1097/XEB.0000000000000282

[43] Pearson A, White H, Bath-Hextall F, Salmond S, Apostolo J, Kirkpatrick P (2015) A mixed-methods approach to systematic reviews. JBI Evid Implement 13:121–131 10.1097/XEB.0000000000000052 26196082

[44] Bossen JK, Hageman MG, King JD, Ring DC (2013) Does rewording MRI reports improve patient understanding and emotional response to a clinical report? Clin Orthop Relat Res 471:3637–3644 23761176 10.1007/s11999-013-3100-x PMC3792273

[45] Norris EC, Halaska C, Sachs PB, Lin C-T, Sanfilippo K, Honce JM (2022) Understanding patient experiences, opinions, and actions taken after viewing their own radiology images online: web-based survey. JMIR Form Res 6:e29496 35468086 10.2196/29496 PMC9086874

[46] Recht MP, Westerhoff M, Doshi AM et al (2022) Video radiology reports: a valuable tool to improve patient-centered radiology. AJR Am J Roentgenol 219:509–519 35441532 10.2214/AJR.22.27512

[47] Short RG, Middleton D, Befera NT, Gondalia R, Tailor TD (2017) Patient-centered radiology reporting: using online crowdsourcing to assess the effectiveness of a web-based interactive radiology report. J Am Coll Radiol 14:1489–1497 29101973 10.1016/j.jacr.2017.07.027

[48] Perlis N, Finelli A, Lovas M et al (2021) Creating patient-centered radiology reports to empower patients undergoing prostate magnetic resonance imaging. Can Urol Assoc J 15:108 33007175 10.5489/cuaj.6585 PMC8021434

[49] Woo S, Andrieu PC, Abu-Rustum NR et al (2024) Bridging communication gaps between radiologists, referring physicians, and patients through standardized structured cancer imaging reporting: the experience with female pelvic MRI assessment using O-RADS and a simulated cohort patient group. Acad Radiol 31:1388–1397 37661555 10.1016/j.acra.2023.08.005 PMC11206174

[50] Wieland J, Quinn K, Stenger K, Cheng S, Acoba J (2023) Patient understanding of oncologic radiology reports: is access to electronic medical records helpful? J Cancer Educ 38:895–899 35984630 10.1007/s13187-022-02204-5

[51] Alarifi M, Patrick T, Jabour A, Wu M, Luo J (2021) Designing a consumer-friendly radiology report using a patient-centered approach. J Digit Imaging 34:705–716 33903982 10.1007/s10278-021-00448-z PMC8329124

[52] Cho JK, Zafar HM, Cook TS (2020) Use of an online crowdsourcing platform to assess patient comprehension of radiology reports and colloquialisms. AJR Am J Roentgenol 214:1316–1320 32208006 10.2214/AJR.19.22202

[53] Kemp J, Short R, Bryant S, Sample L, Befera N (2022) Patient-friendly radiology reporting—implementation and outcomes. J Am Coll Radiol 19:377–383 35152963 10.1016/j.jacr.2021.10.008

[54] Dabrowiecki A, Sadigh G, Duszak JrR (2020) Chest radiograph reporting: public preferences and perceptions. J Am Coll Radiol 17:1259–1268 32413350 10.1016/j.jacr.2020.04.003

[55] Zhang Z, Citardi D, Wang D, Genc Y, Shan J, Fan X (2021) Patients’ perceptions of using artificial intelligence (AI)-based technology to comprehend radiology imaging data. Health Informatics J 27:14604582211011215 33913359 10.1177/14604582211011215

[56] Johnson AJ, Easterling D, Nelson R, Chen MY, Frankel RM (2012) Access to radiologic reports via a patient portal: clinical simulations to investigate patient preferences. J Am Coll Radiol 9:256–263 22469376 10.1016/j.jacr.2011.12.023

[57] Krahe M, Milligan E, Reilly S (2019) Personal health information in research: perceived risk, trustworthiness and opinions from patients attending a tertiary healthcare facility. J Biomed Inform 95:103222 31176040 10.1016/j.jbi.2019.103222

[58] Goodwin E, Davey A, Green C, Hawton A (2021) What drives differences in preferences for health states between patients and the public? A qualitative investigation of respondents’ thought processes. Soc Sci Med 282:114150 34171703 10.1016/j.socscimed.2021.114150

[59] Powell PA, Karimi M, Rowen D, Devlin N, van Hout B, Brazier JE (2023) Hypothetical versus experienced health state valuation: a qualitative study of adult general public views and preferences. Qual Life Res 32:1187–1197 36422771 10.1007/s11136-022-03304-x PMC10063498

[60] List JA, Gallet CA (2001) What experimental protocol influence disparities between actual and hypothetical stated values? Environ Resour Econ 20:241–254

[61] Murphy JJ, Allen PG, Stevens TH, Weatherhead D (2005) A meta-analysis of hypothetical bias in stated preference valuation. Environ Resour Econ 30:313–325

[62] Little J, Berrens RP (2004) Explaining disparities between actual and hypothetical stated values: further investigation using meta-analysis. Econ Bull 3:1–13

[63] Kang MJ, Rangel A, Camus M, Camerer CF (2011) Hypothetical and real choice differentially activate common valuation areas. J Neurosci 31:461–468 21228156 10.1523/JNEUROSCI.1583-10.2011 PMC6623437

[64] Walters K, Christakis DA, Wright DR (2018) Are Mechanical Turk worker samples representative of health status and health behaviors in the US? PLoS One 13:e0198835 29879207 10.1371/journal.pone.0198835 PMC5991724

[65] Levay KE, Freese J, Druckman JN (2016) The demographic and political composition of Mechanical Turk samples. Sage Open 6:2158244016636433

[66] Mortensen K, Alcalá MG, French MT, Hu T (2018) Self-reported health status differs for Amazon’s Mechanical Turk respondents compared with nationally representative surveys. Med Care 56:211–215 29329148 10.1097/MLR.0000000000000871

[67] Dy GW, Gore JL, Muncey WW, Ellison JS, Merguerian PA (2018) Comparative effectiveness of a pilot patient-centered ultrasound report in the management of hydronephrosis. J Pediatr Urol 14:57.e1–57.e7 29054388 10.1016/j.jpurol.2017.08.014

[68] McCaffery KJ, Ayre J, Dodd R et al (2023) Disparities in public understanding, attitudes, and intentions during the Covid-19 pandemic: the role of health literacy. Inf Serv Use 43:1–13

[69] Badarudeen S, Sabharwal S (2010) Assessing readability of patient education materials. Clin Orthop Relat Res 468:2572–2580 20496023 10.1007/s11999-010-1380-y PMC3049622

[70] Mishra V, Dexter JP (2020) Comparison of readability of official public health information about COVID-19 on websites of international agencies and the governments of 15 countries. JAMA Netw Open 3:e2018033 32809028 10.1001/jamanetworkopen.2020.18033 PMC7435342

[71] Briganti G (2024) How ChatGPT works: a mini review. Eur Arch Otorhinolaryngol 281:1565–1569 37991499 10.1007/s00405-023-08337-7

[72] Tunstall L, Von Werra L, Wolf T (2022) Natural language processing with transformers. O’Reilly Media, Inc., Sebastopol

[73] Butler JJ, Puleo J, Harrington MC et al (2024) From technical to understandable: artificial intelligence large language models improve the readability of knee radiology reports. Knee Surg Sports Traumatol Arthrosc 32:1077–1086 38488217 10.1002/ksa.12133

[74] Bajaj S, Gandhi D, Nayar D (2024) Potential applications and impact of ChatGPT in radiology. Acad Radiol 31:1256–1261 37802673 10.1016/j.acra.2023.08.039

[75] European Society of Radiology (ESR) (2023) ESR paper on structured reporting in radiology—update 2023. Insights Imaging 14:199 37995019 10.1186/s13244-023-01560-0 PMC10667169

[76] Nobel JM, Kok EM, Robben SG (2020) Redefining the structure of structured reporting in radiology. Insights Imaging 11:10 32020396 10.1186/s13244-019-0831-6 PMC7000576

