# Supplementary material for: The impact of different radiology report formats on patient information processing: a systematic review
Source: Eur Radiol. 2024 Nov 15;35(5):2644–57. doi: 10.1007/s00330-024-11165-w (PMC12021958; doi:10.1007/s00330-024-11165-w)
Supplement: Supplementary file 1 — ELECTRONIC SUPPLEMENTARY MATERIAL [file 330_2024_11165_MOESM1_ESM.pdf]

# The impact of different radiology report formats on patient information processing: a systematic review

## ELECTRONIC SUPPLEMENTARY MATERIAL

Eur Radiol (2024) van der Mee FAM, Ottenheijm RPG, Gentry EGS, et al.

**Appendix 1.** PRISM Checklist

| Section and Topic | Item # | Checklist item | Location where item is reported |
|---|---|---|---|
| **TITLE** | | | |
| Title | 1 | Identify the report as a systematic review. | 1 |
| **ABSTRACT** | | | |
| Abstract | 2 | See the PRISMA 2020 for Abstracts checklist. | 1 |
| **INTRODUCTION** | | | |
| Rationale | 3 | Describe the rationale for the review in the context of existing knowledge. | 3 |
| Objectives | 4 | Provide an explicit statement of the objective(s) or question(s) the review addresses. | 3 |
| **METHODS** | | | |
| Eligibility criteria | 5 | Specify the inclusion and exclusion criteria for the review and how studies were grouped for the syntheses. | 4 |
| Information sources | 6 | Specify all databases, registers, websites, organisations, reference lists and other sources searched or consulted to identify studies. Specify the date when each source was last searched or consulted. | 4 |
| Search strategy | 7 | Present the full search strategies for all databases, registers and websites, including any filters and limits used. | Appendix 2 |

| Section and Topic | Item # | Checklist item | Location where item is reported |
|---|---|---|---|
| Selection process | 8 | Specify the methods used to decide whether a study met the inclusion criteria of the review, including how many reviewers screened each record and each report retrieved, whether they worked independently, and if applicable, details of automation tools used in the process. | 4 |
| Data collection process | 9 | Specify the methods used to collect data from reports, including how many reviewers collected data from each report, whether they worked independently, any processes for obtaining or confirming data from study investigators, and if applicable, details of automation tools used in the process. | 4 |
| Data items | 10a | List and define all outcomes for which data were sought. Specify whether all results that were compatible with each outcome domain in each study were sought (e.g. for all measures, time points, analyses), and if not, the methods used to decide which results to collect. | 4-5 |
| | 10b | List and define all other variables for which data were sought (e.g. participant and intervention characteristics, funding sources). Describe any assumptions made about any missing or unclear information. | 4-5 |
| Study risk of bias assessment | 11 | Specify the methods used to assess risk of bias in the included studies, including details of the tool(s) used, how many reviewers assessed each study and whether they worked independently, and if applicable, details of automation tools used in the process. | 5 |
| Effect measures | 12 | Specify for each outcome the effect measure(s) (e.g. risk ratio, mean difference) used in the synthesis or presentation of results. | NA |
| Synthesis methods | 13a | Describe the processes used to decide which studies were eligible for each synthesis (e.g. tabulating the study intervention characteristics and comparing against the planned groups for each synthesis (item #5)). | 4-5 |

| Section and Topic | Item # | Checklist item | Location where item is reported |
|---|---|---|---|
| | 13b | Describe any methods required to prepare the data for presentation or synthesis, such as handling of missing summary statistics, or data conversions. | NA |
| | 13c | Describe any methods used to tabulate or visually display results of individual studies and syntheses. | 4-5 |
| | 13d | Describe any methods used to synthesize results and provide a rationale for the choice(s). If meta-analysis was performed, describe the model(s), method(s) to identify the presence and extent of statistical heterogeneity, and software package(s) used. | 5 |
| | 13e | Describe any methods used to explore possible causes of heterogeneity among study results (e.g. subgroup analysis, meta-regression). | NA |
| | 13f | Describe any sensitivity analyses conducted to assess robustness of the synthesized results. | NA |
| Reporting bias assessment | 14 | Describe any methods used to assess risk of bias due to missing results in a synthesis (arising from reporting biases). | NA |
| Certainty assessment | 15 | Describe any methods used to assess certainty (or confidence) in the body of evidence for an outcome. | NA |
| **RESULTS** | | | |
| Study selection | 16a | Describe the results of the search and selection process, from the number of records identified in the search to the number of studies included in the review, ideally using a flow diagram. | 5, Figure 2 |

| Section and Topic | Item # | Checklist item | Location where item is reported |
| --- | --- | --- | --- |
| | 16b | Cite studies that might appear to meet the inclusion criteria, but which were excluded, and explain why they were excluded. | Figure 2 |
| Study characteristics | 17 | Cite each included study and present its characteristics. | Table 1 |
| Risk of bias in studies | 18 | Present assessments of risk of bias for each included study. | 6, Appendix 3 |
| Results of individual studies | 19 | For all outcomes, present, for each study: (a) summary statistics for each group (where appropriate) and (b) an effect estimate and its precision (e.g. confidence/credible interval), ideally using structured tables or plots. | 5-8, Table 3-4 |
| Results of syntheses | 20a | For each synthesis, briefly summarise the characteristics and risk of bias among contributing studies. | 6, Appendix 3 |
| | 20b | Present results of all statistical syntheses conducted. If meta-analysis was done, present for each the summary estimate and its precision (e.g. confidence/credible interval) and measures of statistical heterogeneity. If comparing groups, describe the direction of the effect. | NA |
| | 20c | Present results of all investigations of possible causes of heterogeneity among study results. | NA |

| Section and Topic | Item # | Checklist item | Location where item is reported |
|---|---|---|---|
| | 20d | Present results of all sensitivity analyses conducted to assess the robustness of the synthesized results. | NA |
| Reporting biases | 21 | Present assessments of risk of bias due to missing results (arising from reporting biases) for each synthesis assessed. | NA |
| Certainty of evidence | 22 | Present assessments of certainty (or confidence) in the body of evidence for each outcome assessed. | NA |
| **DISCUSSION** | | | |
| Discussion | 23a | Provide a general interpretation of the results in the context of other evidence. | 8-10 |
| | 23b | Discuss any limitations of the evidence included in the review. | 9-10 |
| | 23c | Discuss any limitations of the review processes used. | 9-10 |
| | 23d | Discuss implications of the results for practice, policy, and future research. | 8-10 |
| **OTHER INFORMATION** | | | |
| Registration and protocol | 24a | Provide registration information for the review, including register name and registration number, or state that the review was not registered. | 4 |
| | 24b | Indicate where the review protocol can be accessed, or state that a protocol was not prepared. | 4 |
| | 24c | Describe and explain any amendments to information provided at registration or in the protocol. | NA |

| Section and Topic | Item # | Checklist item | Location where item is reported |
|---|---|---|---|
| Support | 25 | Describe sources of financial or non-financial support for the review, and the role of the funders or sponsors in the review. | Disclosures |
| Competing interests | 26 | Declare any competing interests of review authors. | Disclosures |
| Availability of data, code and other materials | 27 | Report which of the following are publicly available and where they can be found: template data collection forms; data extracted from included studies; data used for all analyses; analytic code; any other materials used in the review. | NA |

**Appendix 2.** Search strategy in PubMed, Web of Science, EMBASE, and PsycInfo up to September 30th, 2023.

| Search strategy in PubMed (5,134 hits) | | |
|---|---|---|
| Search #1 | "diagnostic imaging"[MeSH Terms] OR "imag*"[Title/Abstract] OR "radiolog*"[Title/Abstract] OR "radiograph*"[Title/Abstract] OR "roentgenograph*"[Title/Abstract] OR "tomograph*"[Title/Abstract] OR "scan*"[Title/Abstract] OR "MRI"[Title/Abstract] OR "CT"[Title/Abstract] OR "x ray*"[Title/Abstract] OR "echograph*"[Title/Abstract] OR "echotomograph*"[Title/Abstract] OR "ultrasonograph*"[Title/Abstract] OR "ultrasound*"[Title/Abstract] OR "sonograph*"[Title/Abstract] OR "mammograph*"[Title/Abstract] OR "echocardiogra*"[Title/Abstract] | Result # 4,804,500 |
| Search #2 | "result*"[Title/Abstract] OR "report*"[Title/Abstract] OR "finding*"[Title/Abstract] | Result # 16,160,531 |
| Search #3 | "health records, personal"[MeSH Terms] OR "medical records systems, computerized"[MeSH Terms] OR "patient access to records"[MeSH Terms] OR "telemedicine"[MeSH Terms] OR "health record*"[Title/Abstract] OR "online record*"[Title/Abstract] OR "patient record*"[Title/Abstract] OR "computerized medical record*"[Title/Abstract] OR "personal medical record*"[Title/Abstract] OR "online medical record*"[Title/Abstract] OR "electronic medical record*"[Title/Abstract] OR "health portal*"[Title/Abstract] OR "online portal*"[Title/Abstract] OR "patient portal*"[Title/Abstract] OR "web portal*"[Title/Abstract] OR "web based portal*"[Title/Abstract] OR "patient access"[Title/Abstract] OR "patients access"[Title/Abstract] OR "online access"[Title/Abstract] OR "ehealth"[Title/Abstract] OR "online health"[Title/Abstract] OR "telehealth"[Title/Abstract] | Result # 167,771 |

| | | |
|---|---|---|
| Search #4 | "patient education as topic"[MeSH Terms] OR "patient participation"[MeSH Terms] OR "self-efficacy"[MeSH Terms] OR "health engagement"[Title/Abstract] OR "patient participation"[Title/Abstract] OR "patients participation"[Title/Abstract] OR "patient involvement"[Title/Abstract] OR "patients involvement"[Title/Abstract] OR "patient activation"[Title/Abstract] OR "patients activation"[Title/Abstract] OR "patient engagement"[Title/Abstract] OR "patients engagement"[Title/Abstract] OR "patient empowerment"[Title/Abstract] OR "patients empowerment"[Title/Abstract] OR "education"[Title/Abstract] OR "perception"[Title/Abstract] OR "comprehens*"[Title/Abstract] OR "satisfaction"[Title/Abstract] OR "experienc*"[Title/Abstract] OR "understand*"[Title/Abstract] OR "interpret*"[Title/Abstract] OR "self-efficacy"[Title/Abstract] OR "information needs"[Title/Abstract] OR "decision"[Title/Abstract] OR "behavioral intention*"[Title/Abstract] OR "action"[Title/Abstract] OR "health behavior"[Title/Abstract] OR "memory"[Title/Abstract] OR "recall"[Title/Abstract] | Result # 5,678,192 |
| Search #5 | **Search #1 AND Search #2**<br><br>("diagnostic imaging"[MeSH Terms] OR "imag*"[Title/Abstract] OR "radiolog*"[Title/Abstract] OR "radiograph*"[Title/Abstract] OR "roentgenograph*"[Title/Abstract] OR "tomograph*"[Title/Abstract] OR "scan*"[Title/Abstract] OR "MRI"[Title/Abstract] OR "CT"[Title/Abstract] OR "x ray*"[Title/Abstract] OR "echograph*"[Title/Abstract] OR "echotomograph*"[Title/Abstract] OR "ultrasonograph*"[Title/Abstract] OR "ultrasound*"[Title/Abstract] OR "sonograph*"[Title/Abstract] OR "mammograph*"[Title/Abstract] | Result # 2,808,603 |

| | | |
|---|---|---|
| | OR "echocardiogra*"[Title/Abstract]) AND ("result*"[Title/Abstract] OR "report*"[Title/Abstract] OR "finding*"[Title/Abstract]) | |
| Search #6 | **Search #5 AND Search #3**<br><br>("diagnostic imaging"[MeSH Terms] OR "imag*"[Title/Abstract] OR "radiolog*"[Title/Abstract] OR "radiograph*"[Title/Abstract] OR "roentgenograph*"[Title/Abstract] OR "tomograph*"[Title/Abstract] OR "scan*"[Title/Abstract] OR "MRI"[Title/Abstract] OR "CT"[Title/Abstract] OR "x ray*"[Title/Abstract] OR "echograph*"[Title/Abstract] OR "echotomograph*"[Title/Abstract] OR "ultrasonograph*"[Title/Abstract] OR "ultrasound*"[Title/Abstract] OR "sonograph*"[Title/Abstract] OR "mammograph*"[Title/Abstract] OR "echocardiogra*"[Title/Abstract]) AND ("result*"[Title/Abstract] OR "report*"[Title/Abstract] OR "finding*"[Title/Abstract]) AND ("health records, personal"[MeSH Terms] OR "medical records systems, computerized"[MeSH Terms] OR "patient access to records"[MeSH Terms] OR "telemedicine"[MeSH Terms] OR "health record*"[Title/Abstract] OR "online record*"[Title/Abstract] OR "patient record*"[Title/Abstract] OR "computerized medical record*"[Title/Abstract] OR "personal medical record*"[Title/Abstract] OR "online medical record*"[Title/Abstract] OR "electronic medical record*"[Title/Abstract] OR "health portal*"[Title/Abstract] OR "online portal*"[Title/Abstract] OR "patient portal*"[Title/Abstract] OR "web portal*"[Title/Abstract] OR "web based portal*"[Title/Abstract] OR "patient access"[Title/Abstract] OR "patients access"[Title/Abstract] OR "online access"[Title/Abstract] OR "ehealth"[Title/Abstract] OR "online health"[Title/Abstract] OR "telehealth"[Title/Abstract]) | Result # 12,972 |

| | | |
|---|---|---|
| Search #7 | **Search #6 AND Search #4**<br><br>("diagnostic imaging"[MeSH Terms] OR "imag*"[Title/Abstract] OR "radiolog*"[Title/Abstract] OR "radiograph*"[Title/Abstract] OR "roentgenograph*"[Title/Abstract] OR "tomograph*"[Title/Abstract] OR "scan*"[Title/Abstract] OR "MRI"[Title/Abstract] OR "CT"[Title/Abstract] OR "x ray*"[Title/Abstract] OR "echograph*"[Title/Abstract] OR "echotomograph*"[Title/Abstract] OR "ultrasonograph*"[Title/Abstract] OR "ultrasound*"[Title/Abstract] OR "sonograph*"[Title/Abstract] OR "mammograph*"[Title/Abstract] OR "echocardiogra*"[Title/Abstract]) AND ("result*"[Title/Abstract] OR "report*"[Title/Abstract] OR "finding*"[Title/Abstract]) AND ("health records, personal"[MeSH Terms] OR "medical records systems, computerized"[MeSH Terms] OR "patient access to records"[MeSH Terms] OR "telemedicine"[MeSH Terms] OR "health record*"[Title/Abstract] OR "online record*"[Title/Abstract] OR "patient record*"[Title/Abstract] OR "computerized medical record*"[Title/Abstract] OR "personal medical record*"[Title/Abstract] OR "online medical record*"[Title/Abstract] OR "electronic medical record*"[Title/Abstract] OR "health portal*"[Title/Abstract] OR "online portal*"[Title/Abstract] OR "patient portal*"[Title/Abstract] OR "web portal*"[Title/Abstract] OR "web based portal*"[Title/Abstract] OR "patient access"[Title/Abstract] OR "patients access"[Title/Abstract] OR "online access"[Title/Abstract] OR "ehealth"[Title/Abstract] OR "online health"[Title/Abstract] OR "telehealth"[Title/Abstract]) AND ("patient education as topic"[MeSH Terms] OR "patient participation"[MeSH Terms] OR "self-efficacy"[MeSH Terms] OR "health engagement"[Title/Abstract] OR "patient participation"[Title/Abstract] OR "patients participation"[Title/Abstract] OR "patient involvement"[Title/Abstract] | Result # 5,134 |

| | | |
|---|---|---|
| | OR "patients involvement"[Title/Abstract] OR "patient activation"[Title/Abstract] OR "patients activation"[Title/Abstract] OR "patient engagement"[Title/Abstract] OR "patients engagement"[Title/Abstract] OR "patient empowerment"[Title/Abstract] OR "patients empowerment"[Title/Abstract] OR "education"[Title/Abstract] OR "perception"[Title/Abstract] OR "comprehens*"[Title/Abstract] OR "satisfaction"[Title/Abstract] OR "experienc*"[Title/Abstract] OR "understand*"[Title/Abstract] OR "interpret*"[Title/Abstract] OR "self-efficacy"[Title/Abstract] OR "information needs"[Title/Abstract] OR "decision"[Title/Abstract] OR "behavioral intention*"[Title/Abstract] OR "action"[Title/Abstract] OR "health behavior"[Title/Abstract] OR "memory"[Title/Abstract] OR "recall"[Title/Abstract]) | |

| Search strategy in Web of Science (3,471 hits) | | |
|---|---|---|
| Search #1 | TS= "imag*" OR TS= "radiolog*" OR TS= "radiograph*" OR TS= "roentgenograph*" OR TS= "tomograph*" OR TS= "scan*" OR TS= "MRI" OR TS= "CT" OR TS= "x ray*" OR TS= "echograph*" OR TS= "echotomograph*" OR TS= "ultrasonograph*" OR TS= "ultrasound*" OR TS= "sonograph*" OR TS= "mammograph*" OR TS= "echocardiogra*" | Result # 6,670,205 |
| Search #2 | TS= "result*" OR TS= "report*" OR TS= "finding*" | Result # 26,212,691 |
| Search #3 | TS= "health record*" OR TS= "online record*" OR TS= "patient record*" OR TS= "computerized medical record*" OR TS= "personal medical record*" OR TS= "online medical record*" OR TS= "electronic medical record*" OR TS= "health portal*" OR TS= "online portal*" OR TS= "patient portal*" OR TS= "web portal*" OR TS= "web based portal*" OR TS= "patient access" OR TS= "patients access" OR TS= "online access" OR TS= "ehealth" OR TS= "online health" OR TS= "telehealth" | Result # 118,722 |
| Search #4 | TS= "health engagement" OR TS= "patient participation" OR TS= "patients participation" OR TS= "patient involvement" OR TS= "patients involvement" OR TS= "patient activation" OR TS= "patients activation" OR TS= "patient engagement" OR TS= "patients engagement" OR TS= "patient empowerment" OR TS= "patients empowerment" OR TS= "education" OR TS= "perception" OR TS= "comprehens*" OR TS= "satisfaction" OR TS= "experienc*" OR TS= "understand*" OR TS= "interpret*" OR TS= "self-efficacy" OR TS= "information needs" OR TS= "decision" OR TS= "behavioral | Result # 10,366,781 |

| | | |
|---|---|---|
| | intention*" OR TS= "action" OR TS= "health behavior" OR TS= "memory" OR TS= "recall" | |
| Search #5 | **Search #1 AND Search #2**<br><br>(TS= "imag*" OR TS= "radiolog*" OR TS= "radiograph*" OR TS= "roentgenograph*" OR TS= "tomograph*" OR TS= "scan*" OR TS= "MRI" OR TS= "CT" OR TS= "x ray*" OR TS= "echograph*" OR TS= "echotomograph*" OR TS= "ultrasonograph*" OR TS= "ultrasound*" OR TS= "sonograph*" OR TS= "mammograph*" OR TS= "echocardiogra*") AND (TS= "result*" OR TS= "report*" OR TS= "finding*") | Result # 3,689,396 |
| Search #6 | **Search #5 AND Search #3**<br><br>(TS= "imag*" OR TS= "radiolog*" OR TS= "radiograph*" OR TS= "roentgenograph*" OR TS= "tomograph*" OR TS= "scan*" OR TS= "MRI" OR TS= "CT" OR TS= "x ray*" OR TS= "echograph*" OR TS= "echotomograph*" OR TS= "ultrasonograph*" OR TS= "ultrasound*" OR TS= "sonograph*" OR TS= "mammograph*" OR TS= "echocardiogra*") AND (TS= "result*" OR TS= "report*" OR TS= "finding*") AND (TS= "health record*" OR TS= "online record*" OR TS= "patient record*" OR TS= "computerized medical record*" OR TS= "personal medical record*" OR TS= "online medical record*" OR TS= "electronic medical record*" OR TS= "health portal*" OR TS= "online portal*" OR TS= "patient portal*" OR TS= "web portal*" OR TS= "web based portal*" OR TS= "patient access" OR TS= "patients access" OR TS= "online access" OR TS= "ehealth" OR TS= "online health" OR TS= "telehealth") | Result # 8,728 |

| | | |
|---|---|---|
| Search #7 | **Search #6 AND Search #4**<br><br>(TS= "imag*" OR TS= "radiolog*" OR TS= "radiograph*" OR TS= "roentgenograph*" OR TS= "tomograph*" OR TS= "scan*" OR TS= "MRI" OR TS= "CT" OR TS= "x ray*" OR TS= "echograph*" OR TS= "echotomograph*" OR TS= "ultrasonograph*" OR TS= "ultrasound*" OR TS= "sonograph*" OR TS= "mammograph*" OR TS= "echocardiogra*") AND (TS= "result*" OR TS= "report*" OR TS= "finding*") AND (TS= "health record*" OR TS= "online record*" OR TS= "patient record*" OR TS= "computerized medical record*" OR TS= "personal medical record*" OR TS= "online medical record*" OR TS= "electronic medical record*" OR TS= "health portal*" OR TS= "online portal*" OR TS= "patient portal*" OR TS= "web portal*" OR TS= "web based portal*" OR TS= "patient access" OR TS= "patients access" OR TS= "online access" OR TS= "ehealth" OR TS= "online health" OR TS= "telehealth") AND (TS= "health engagement" OR TS= "patient participation" OR TS= "patients participation" OR TS= "patient involvement" OR TS= "patients involvement" OR TS= "patient activation" OR TS= "patients activation" OR TS= "patient engagement" OR TS= "patients engagement" OR TS= "patient empowerment" OR TS= "patients empowerment" OR TS= "education" OR TS= "perception" OR TS= "comprehens*" OR TS= "satisfaction" OR TS= "experienc*" OR TS= "understand*" OR TS= "interpret*" OR TS= "self-efficacy" OR TS= "information needs" OR TS= "decision" OR TS= "behavioral intention*" OR TS= "action" OR TS= "health behavior" OR TS= "memory" OR TS= "recall") | Result # 3,471 |

| Search strategy in EMBASE (2,265 hits) | | |
|---|---|---|
| Search #1 | exp diagnostic imaging/ OR exp radiology/ OR exp radiography/ OR exp tomography/ OR exp nuclear magnetic resonance imaging/ OR exp computer assisted tomography/ OR exp x-ray computed tomography/ OR exp x-ray/ OR exp echograph/ OR exp echography/ OR exp ultrasound/ OR exp mammography/ OR imag*.ti,ab,kf. OR radiolog*.ti,ab,kf. OR radiograph*.ti,ab,kf. OR roentgenograph*.ti,ab,kf. OR tomograph*.ti,ab,kf. OR scan*.ti,ab,kf. OR MRI.ti,ab,kf. OR CT.ti,ab,kf. OR x ray*.ti,ab,kf. OR echograph*.ti,ab,kf. OR echotomograph*.ti,ab,kf. OR ultrasonograph*.ti,ab,kf. OR ultrasound*.ti,ab,kf. OR sonograph*.ti,ab,kf. OR mammograph*.ti,ab,kf. OR echocardiogra*.ti,ab,kf. | Result # 6,104,186 |
| Search #2 | result*.ti,ab,kf. OR report*.ti,ab,kf. OR finding*.ti,ab,kf. | Result # 21,082,806 |
| Search #3 | exp electronic health record/ OR exp electronic medical record/ OR exp medical record/ OR exp access to information/ OR exp telehealth/ OR health record*.ti,ab,kf. OR online record*.ti,ab,kf. OR patient record*.ti,ab,kf. OR computerized medical record*.ti,ab,kf. OR personal medical record*.ti,ab,kf. OR online medical record*.ti,ab,kf. OR electronic medical record*.ti,ab,kf. OR health portal*.ti,ab,kf. OR online portal*.ti,ab,kf. OR patient portal*.ti,ab,kf. OR web portal*.ti,ab,kf. OR web based portal*.ti,ab,kf. OR patient access.ti,ab,kf. OR patients access.ti,ab,kf. OR online access.ti,ab,kf. OR ehealth.ti,ab,kf. OR online health.ti,ab,kf. OR telehealth.ti,ab,kf. | Result # 487,825 |
| Search #4 | exp patient participation/ or exp patient engagement/ or exp patient empowerment/ or exp patient education/ or exp perception/ or exp | Result # 7,641,650 |

| | | |
|---|---|---|
| | comprehension/ or exp satisfaction/ or exp patient satisfaction/ or exp experience/ or exp self concept/ or health engagement.ti,ab,kf. or patient participation.ti,ab,kf. or patients participation.ti,ab,kf. or patient involvement.ti,ab,kf. or patients involvement.ti,ab,kf. or patient activation.ti,ab,kf. or patients activation.ti,ab,kf. or patient engagement.ti,ab,kf. or patients engagement.ti,ab,kf. or patient empowerment.ti,ab,kf. or patients empowerment.ti,ab,kf. or education.ti,ab,kf. or perception.ti,ab,kf. or comprehens*.ti,ab,kf. or satisfaction.ti,ab,kf. or experienc*.ti,ab,kf. or understand*.ti,ab,kf. or interpret*.ti,ab,kf. or self-efficacy.ti,ab,kf. or self concept.ti,ab,kf. or information needs.ti,ab,kf. or decision.ti,ab,kf. or behavioral intention.ti,ab,kf. or action.ti,ab,kf. or health behavior.ti,ab,kf. or memory.ti,ab,kf. or recall.ti,ab,kf. | |
| Search #5 | **Search #1 AND Search #2**<br><br>(exp diagnostic imaging/ OR exp radiology/ OR exp radiography/ OR exp tomography/ OR exp nuclear magnetic resonance imaging/ OR exp computer assisted tomography/ OR exp x-ray computed tomography/ OR exp x-ray/ OR exp echograph/ OR exp echography/ OR exp ultrasound/ OR exp mammography/ OR imag*.ti,ab,kf. OR radiolog*.ti,ab,kf. OR radiograph*.ti,ab,kf. OR roentgenograph*.ti,ab,kf. OR tomograph*.ti,ab,kf. OR scan*.ti,ab,kf. OR MRI.ti,ab,kf. OR CT.ti,ab,kf. OR x ray*.ti,ab,kf. OR echograph*.ti,ab,kf. OR echotomograph*.ti,ab,kf. OR ultrasonograph*.ti,ab,kf. OR ultrasound*.ti,ab,kf. OR sonograph*.ti,ab,kf. OR mammograph*.ti,ab,kf. OR echocardiogra*.ti,ab,kf.) AND (result*.ti,ab,kf. OR report*.ti,ab,kf. OR finding*.ti,ab,kf.) | Result # 4,052,724 |

| | | |
|---|---|---|
| Search #6 | **Search #5 AND Search #3**<br><br>(exp diagnostic imaging/ OR exp radiology/ OR exp radiography/ OR exp tomography/ OR exp nuclear magnetic resonance imaging/ OR exp computer assisted tomography/ OR exp x-ray computed tomography/ OR exp x-ray/ OR exp echograph/ OR exp echography/ OR exp ultrasound/ OR exp mammography/ OR imag*.ti,ab,kf. OR radiolog*.ti,ab,kf. OR radiograph*.ti,ab,kf. OR roentgenograph*.ti,ab,kf. OR tomograph*.ti,ab,kf. OR scan*.ti,ab,kf. OR MRI.ti,ab,kf. OR CT.ti,ab,kf. OR x ray*.ti,ab,kf. OR echograph*.ti,ab,kf. OR echotomograph*.ti,ab,kf. OR ultrasonograph*.ti,ab,kf. OR ultrasound*.ti,ab,kf. OR sonograph*.ti,ab,kf. OR mammograph*.ti,ab,kf. OR echocardiogra*.ti,ab,kf.) AND (result*.ti,ab,kf. OR report*.ti,ab,kf. OR finding*.ti,ab,kf.) AND (exp electronic health record/ OR exp electronic medical record/ OR exp medical record/ OR exp access to information/ OR exp telehealth/ OR health record*.ti,ab,kf. OR online record*.ti,ab,kf. OR patient record*.ti,ab,kf. OR computerized medical record*.ti,ab,kf. OR personal medical record*.ti,ab,kf. OR online medical record*.ti,ab,kf. OR electronic medical record*.ti,ab,kf. OR health portal*.ti,ab,kf. OR online portal*.ti,ab,kf. OR patient portal*.ti,ab,kf. OR web portal*.ti,ab,kf. OR web based portal*.ti,ab,kf. OR patient access.ti,ab,kf. OR patients access.ti,ab,kf. OR online access.ti,ab,kf. OR ehealth.ti,ab,kf. OR online health.ti,ab,kf. OR telehealth.ti,ab,kf.) | Result # 63,109 |
| Search #7 | **Search #6 AND Search #4**<br><br>(exp diagnostic imaging/ OR exp radiology/ OR exp radiography/ OR exp tomography/ OR exp nuclear magnetic resonance imaging/ OR | Result # 20,764 |

| | | |
|---|---|---|
| | exp computer assisted tomography/ OR exp x-ray computed tomography/ OR exp x-ray/ OR exp echograph/ OR exp echography/ OR exp ultrasound/ OR exp mammography/ OR imag*.ti,ab,kf. OR radiolog*.ti,ab,kf. OR radiograph*.ti,ab,kf. OR roentgenograph*.ti,ab,kf. OR tomograph*.ti,ab,kf. OR scan*.ti,ab,kf. OR MRI.ti,ab,kf. OR CT.ti,ab,kf. OR x ray*.ti,ab,kf. OR echograph*.ti,ab,kf. OR echotomograph*.ti,ab,kf. OR ultrasonograph*.ti,ab,kf. OR ultrasound*.ti,ab,kf. OR sonograph*.ti,ab,kf. OR mammograph*.ti,ab,kf. OR echocardiogra*.ti,ab,kf.) AND (result*.ti,ab,kf. OR report*.ti,ab,kf. OR finding*.ti,ab,kf.) AND (exp electronic health record/ OR exp electronic medical record/ OR exp medical record/ OR exp access to information/ OR exp telehealth/ OR health record*.ti,ab,kf. OR online record*.ti,ab,kf. OR patient record*.ti,ab,kf. OR computerized medical record*.ti,ab,kf. OR personal medical record*.ti,ab,kf. OR online medical record*.ti,ab,kf. OR electronic medical record*.ti,ab,kf. OR health portal*.ti,ab,kf. OR online portal*.ti,ab,kf. OR patient portal*.ti,ab,kf. OR web portal*.ti,ab,kf. OR web based portal*.ti,ab,kf. OR patient access.ti,ab,kf. OR patients access.ti,ab,kf. OR online access.ti,ab,kf. OR ehealth.ti,ab,kf. OR online health.ti,ab,kf. OR telehealth.ti,ab,kf.) AND (exp patient participation/ or exp patient engagement/ or exp patient empowerment/ or exp patient education/ or exp perception/ or exp comprehension/ or exp satisfaction/ or exp patient satisfaction/ or exp experience/ or exp self concept/ or health engagement.ti,ab,kf. or patient participation.ti,ab,kf. or patients participation.ti,ab,kf. or patient involvement.ti,ab,kf. or patients involvement.ti,ab,kf. or patient activation.ti,ab,kf. or patients activation.ti,ab,kf. or patient engagement.ti,ab,kf. or patients engagement.ti,ab,kf. or patient empowerment.ti,ab,kf. or patients empowerment.ti,ab,kf. or education.ti,ab,kf. or perception.ti,ab,kf. or comprehens*.ti,ab,kf. or satisfaction.ti,ab,kf. or experienc*.ti,ab,kf. or | |

| | | |
|---|---|---|
| | understand*.ti,ab,kf. or interpret*.ti,ab,kf. or self-efficacy.ti,ab,kf. or self concept.ti,ab,kf. or information needs.ti,ab,kf. or decision.ti,ab,kf. or behavioral intention.ti,ab,kf. or action.ti,ab,kf. or health behavior.ti,ab,kf. or memory.ti,ab,kf. or recall.ti,ab,kf.) | |
| Search #8 | **Search #7**<br><br>Filters: remove preprint records AND exclude MEDLINE citations | Results # 2,265 |

| Search strategy in PsycInfo (172 hits) | | |
|---|---|---|
| Search #1 | DE ("Radiology" OR "Roentgenography" OR "Mammography" OR "Tomography" OR "Magnetic Resonance Imaging" OR "Single Photon Emission Computed Tomography" OR "Ultrasound") OR TI ("imag*" OR "radiolog*" OR "radiograph*" OR "roentgenograph*" OR "tomograph*" OR "scan*" OR "MRI" OR "CT" OR "x ray*" OR "echograph*" OR "echotomograph*" OR "ultrasonograph*" OR "ultrasound*" OR "sonograph*" OR "mammograph*" OR "echocardiogra*") OR AB ("imag*" OR "radiolog*" OR "radiograph*" OR "roentgenograph*" OR "tomograph*" OR "scan*" OR "MRI" OR "CT" OR "x ray*" OR "echograph*" OR "echotomograph*" OR "ultrasonograph*" OR "ultrasound*" OR "sonograph*" OR "mammograph*" OR "echocardiogra*") | Result # 326,281 |
| Search #2 | TI ("result*" OR "report*" OR "finding*") OR AB ("result*" OR "report*" OR "finding*") | Result # 2,886,748 |
| Search #3 | DE ("Medical Records" OR "Client Records" OR "Electronic Health Records") OR TI ("health record*" OR "online record*" OR "patient record*" OR "computerized medical record*" OR "personal medical record*" OR "online medical record*" OR "electronic medical record*" OR "health portal*" OR "online portal*" OR "patient portal*" OR "web portal*" OR "web based portal*" OR "patient access" OR "patients access" OR "online access" OR "ehealth" OR "online health" OR "telehealth") OR AB ("health record*" OR "online record*" OR "patient record*" OR "computerized medical record*" OR "personal medical record*" OR "online medical record*" OR "electronic medical record*" OR "health portal*" OR "online portal*" OR "patient portal*" OR "web portal*" OR "web based portal*" OR "patient access" OR "patients | Result # 19,295 |

| | | |
|---|---|---|
| | access" OR "online access" OR "ehealth" OR "online health" OR "telehealth") | |
| Search #4 | DE ("Client Participation" OR "Client Education" OR "Client Satisfaction" OR "Self-Efficacy" OR "Behavioral Intention" OR "Health Behavior") OR TI ("health engagement" OR "patient participation" OR "patients participation" OR "patient involvement" OR "patients involvement" OR "patient activation" OR "patients activation" OR "patient engagement" OR "patients engagement" OR "patient empowerment" OR "patients empowerment" OR "education" OR "perception" OR "comprehens*" OR "satisfaction" OR "experienc*" OR "understand*" OR "interpret*" OR "self-efficacy" OR "information needs" OR "decision" OR "behavioral intention*" OR "action" OR "health behavior" OR "memory" OR "recall") OR AB ("health engagement" OR "patient participation" OR "patients participation" OR "patient involvement" OR "patients involvement" OR "patient activation" OR "patients activation" OR "patient engagement" OR "patients engagement" OR "patient empowerment" OR "patients empowerment" OR "education" OR "perception" OR "comprehens*" OR "satisfaction" OR "experienc*" OR "understand*" OR "interpret*" OR "self-efficacy" OR "information needs" OR "decision" OR "behavioral intention*" OR "action" OR "health behavior" OR "memory" OR "recall") | Result # 2,367,047 |
| Search #5 | **Search #1 AND Search #2**<br><br>(DE ("Radiology" OR "Roentgenography" OR "Mammography" OR "Tomography" OR "Magnetic Resonance Imaging" OR "Single Photon Emission Computed Tomography" OR "Ultrasound") OR TI ("imag*" OR "radiolog*" OR "radiograph*" OR "roentgenograph*" OR "tomograph*" OR "scan*" OR "MRI" OR "CT" OR "x ray*" OR "echograph*" OR | Result # 203,304 |

| | | |
|---|---|---|
| | "echotomograph*" OR "ultrasonograph*" OR "ultrasound*" OR "sonograph*" OR "mammograph*" OR "echocardiogra*") OR AB ("imag*" OR "radiolog*" OR "radiograph*" OR "roentgenograph*" OR "tomograph*" OR "scan*" OR "MRI" OR "CT" OR "x ray*" OR "echograph*" OR "echotomograph*" OR "ultrasonograph*" OR "ultrasound*" OR "sonograph*" OR "mammograph*" OR "echocardiogra*")) AND (TI ("result*" OR "report*" OR "finding*") OR AB ("result*" OR "report*" OR "finding*")) | |
| Search #6 | **Search #5 AND Search #3**<br><br>(DE ("Radiology" OR "Roentgenography" OR "Mammography" OR "Tomography" OR "Magnetic Resonance Imaging" OR "Single Photon Emission Computed Tomography" OR "Ultrasound") OR TI ("imag*" OR "radiolog*" OR "radiograph*" OR "roentgenograph*" OR "tomograph*" OR "scan*" OR "MRI" OR "CT" OR "x ray*" OR "echograph*" OR "echotomograph*" OR "ultrasonograph*" OR "ultrasound*" OR "sonograph*" OR "mammograph*" OR "echocardiogra*") OR AB ("imag*" OR "radiolog*" OR "radiograph*" OR "roentgenograph*" OR "tomograph*" OR "scan*" OR "MRI" OR "CT" OR "x ray*" OR "echograph*" OR "echotomograph*" OR "ultrasonograph*" OR "ultrasound*" OR "sonograph*" OR "mammograph*" OR "echocardiogra*")) AND (TI ("result*" OR "report*" OR "finding*") OR AB ("result*" OR "report*" OR "finding*")) AND (DE ("Medical Records" OR "Client Records" OR "Electronic Health Records") OR TI ("health record*" OR "online record*" OR "patient record*" OR "computerized medical record*" OR "personal medical record*" OR "online medical record*" OR "electronic medical record*" OR "health portal*" OR "online portal*" OR "patient portal*" OR "web portal*" OR "web based portal*" OR "patient access" OR "patients access" OR "online access" | Result # 464 |

| | | |
|---|---|---|
| | OR "ehealth" OR "online health" OR "telehealth") OR AB ("health record*" OR "online record*" OR "patient record*" OR "computerized medical record*" OR "personal medical record*" OR "online medical record*" OR "electronic medical record*" OR "health portal*" OR "online portal*" OR "patient portal*" OR "web portal*" OR "web based portal*" OR "patient access" OR "patients access" OR "online access" OR "ehealth" OR "online health" OR "telehealth")) | |
| Search #7 | **Search #6 AND Search #4**<br><br>(DE ("Radiology" OR "Roentgenography" OR "Mammography" OR "Tomography" OR "Magnetic Resonance Imaging" OR "Single Photon Emission Computed Tomography" OR "Ultrasound") OR TI ("imag*" OR "radiolog*" OR "radiograph*" OR "roentgenograph*" OR "tomograph*" OR "scan*" OR "MRI" OR "CT" OR "x ray*" OR "echograph*" OR "echotomograph*" OR "ultrasonograph*" OR "ultrasound*" OR "sonograph*" OR "mammograph*" OR "echocardiogra*") OR AB ("imag*" OR "radiolog*" OR "radiograph*" OR "roentgenograph*" OR "tomograph*" OR "scan*" OR "MRI" OR "CT" OR "x ray*" OR "echograph*" OR "echotomograph*" OR "ultrasonograph*" OR "ultrasound*" OR "sonograph*" OR "mammograph*" OR "echocardiogra*")) AND (TI ("result*" OR "report*" OR "finding*") OR AB ("result*" OR "report*" OR "finding*")) AND (DE ("Medical Records" OR "Client Records" OR "Electronic Health Records") OR TI ("health record*" OR "online record*" OR "patient record*" OR "computerized medical record*" OR "personal medical record*" OR "online medical record*" OR "electronic medical record*" OR "health portal*" OR "online portal*" OR "patient portal*" OR "web portal*" OR "web based portal*" OR "patient access" OR "patients access" OR "online access" OR "ehealth" OR "online health" OR "telehealth") OR AB ("health | Result # 211 |

| | | |
|---|---|---|
| | record*" OR "online record*" OR "patient record*" OR "computerized medical record*" OR "personal medical record*" OR "online medical record*" OR "electronic medical record*" OR "health portal*" OR "online portal*" OR "patient portal*" OR "web portal*" OR "web based portal*" OR "patient access" OR "patients access" OR "online access" OR "ehealth" OR "online health" OR "telehealth")) AND (DE ("Client Participation" OR "Client Education" OR "Client Satisfaction" OR "Self-Efficacy" OR "Behavioral Intention" OR "Health Behavior") OR TI ("health engagement" OR "patient participation" OR "patients participation" OR "patient involvement" OR "patients involvement" OR "patient activation" OR "patients activation" OR "patient engagement" OR "patients engagement" OR "patient empowerment" OR "patients empowerment" OR "education" OR "perception" OR "comprehens*" OR "satisfaction" OR "experienc*" OR "understand*" OR "interpret*" OR "self-efficacy" OR "information needs" OR "decision" OR "behavioral intention*" OR "action" OR "health behavior" OR "memory" OR "recall") OR AB ("health engagement" OR "patient participation" OR "patients participation" OR "patient involvement" OR "patients involvement" OR "patient activation" OR "patients activation" OR "patient engagement" OR "patients engagement" OR "patient empowerment" OR "patients empowerment" OR "education" OR "perception" OR "comprehens*" OR "satisfaction" OR "experienc*" OR "understand*" OR "interpret*" OR "self-efficacy" OR "information needs" OR "decision" OR "behavioral intention*" OR "action" OR "health behavior" OR "memory" OR "recall")) | |
| Search #8 | **Search #7**<br><br>Filters: remove dissertations | Result # 172 |

**Appendix 3.** Detailed overview of quality assessment of all included studies (n = 18).

| **Author (year)** | **Score[a]** | **Criteria from the Mixed Methods Appraisal Tool** | | | | | | | | | | | | | | | | | | | | | | | | | | |
|---|---|---|---|---|---|---|---|---|---|---|---|---|---|---|---|---|---|---|---|---|---|---|---|---|---|---|---|---|
| | | **S1** | **S2** | **1.1** | **1.2** | **1.3** | **1.4** | **1.5** | **2.1** | **2.2** | **2.3** | **2.4** | **2.5** | **3.1** | **3.2** | **3.3** | **3.4** | **3.5** | **4.1** | **4.2** | **4.3** | **4.4** | **4.5** | **5.1** | **5.2** | **5.3** | **5.4** | **5.5** |
| **Alarifi et al. (2021)** | **** | Y | Y | | | | | | Y | Y | Y | C | Y | | | | | | | | | | | | | | | |
| **Bossen et al. (2013)** | *** | Y | Y | | | | | | | | | | | N | Y | Y | C | Y | | | | | | | | | | |
| **Cho et al. (2020)** | ** | Y | Y | | | | | | | | | | | N | C | C | Y | Y | | | | | | | | | | |
| **Cook et al. (2017)** | ** | Y | Y | | | | | | | | | | | C | C | Y | C | Y | | | | | | | | | | |
| **Dabrowiecki et al. (2020)** | ** | Y | Y | | | | | | | | | | | | | | | | Y | N | C | C | Y | | | | | |
| **Dy et al. (2018)** | *** | Y | Y | | | | | | Y | Y | N | C | Y | | | | | | | | | | | | | | | |
| **Gunn et al. (2017)** | * | Y | Y | | | | | | | | | | | N | C | C | N | Y | | | | | | | | | | |
| **Johnson et al. (2012)** | *** | Y | Y | | | | | | | | | | | | | | | | Y | N | N | Y | Y | | | | | |
| **Kadom et al. (2021)** | *** | Y | Y | | | | | | | | | | | | | | | | Y | N | C | Y | Y | | | | | |
| **Kemp et al. (2022)** | * | Y | Y | | | | | | | | | | | C | N | C | C | Y | | | | | | | | | | |

| | | | | | | | | | | | | | | | | | | | | | | | | | | | | |
|---|---|---|---|---|---|---|---|---|---|---|---|---|---|---|---|---|---|---|---|---|---|---|---|---|---|---|---|---|
| **Norris et al. (2022)** | *** | Y | Y | | | | | | | | | | | | | | | | | Y | N | C | Y | Y | | | | | |
| **Perlis et al. (2021)** | ***** | Y | Y | Y | Y | Y | Y | Y | | | | | | | | | | | | | | | | | | | | | |
| **Perlis et al. (2022)** | ** | Y | Y | | | | | | C | Y | C | C | Y | | | | | | | | | | | | | | | | |
| **Recht et al. (2022)** | ** | Y | Y | | | | | | | | | | | C | C | Y | C | Y | | | | | | | | | | | |
| **Short et al. (2017)** | ** | Y | Y | | | | | | C | C | Y | C | Y | | | | | | | | | | | | | | | | |
| **Wieland et al. (2023)** | ** | Y | Y | | | | | | C | Y | C | C | Y | | | | | | | | | | | | | | | | |
| **Woo et al. (2023)** | ** | Y | Y | | | | | | | | | | | | | | | | | Y | C | C | C | Y | | | | | |
| **Zhang et al. (2021)** | ***** | Y | Y | Y | Y | Y | Y | Y | | | | | | | | | | | | | | | | | | | | | |

*[a]Score was calculated as follows: one star was awarded for each ‘Y’, while ‘N’ or ‘C’ did not acquire any stars.*

*Abbreviations: Y, yes; N, no; C, can’t tell.*

**Appendix 4.** Overview of all outcomes measures included in the categories of patient information processing (perception, decision, action, and memory).

Perception – affective perception

- Anxiety [28, 29, 44, 46, 47]
- Calmness [44]
- Feeling in control [44].
- Pleasure [44].
- Reassurance [45].
- Confusion [45].
- Worry [45].

Perception – perceived magnitude

- Sense to contact physician immediately [29].
- Clarity of the chance of cancer [48, 49].

Perception – cognitive perception

- Self-reported level of understanding [15, 27, 28, 44-46, 50-54]
- Self-reported ease of reading [49, 50].
- Familiarity with medical terms [50].
- Objective level of understanding [47, 51, 52].

Perception – perception of communication

- Perceived ease of use [51].
- Satisfaction [44, 49, 51].
- Helpfulness [15, 46].

- Usefulness [28, 55, 67].
- Experience [28, 45-47, 53].
- Preferred report format [29, 46, 48, 51, 56].

Decision/behavioral intention

- Most likely follow-up action/ next step [29, 54].
- Use of educational resources for better understanding [56].

Action/actual health behavior

- With whom were questions after viewing the report discussed [45].
- What was done with radiology images [45].
- Behavior after viewing report [28].

Memory

None of the included studies assessed memory as outcome measure.
